# Spin-Coating Fabrication Method of PDMS/NdFeB Composites Using Chitosan/PCL Coating

**DOI:** 10.3390/ma17091973

**Published:** 2024-04-24

**Authors:** Anna Powojska, Arkadiusz Mystkowski, Edison Gundabattini, Joanna Mystkowska

**Affiliations:** 1 Department of Biomaterials and Medical Devices, Institute of Biomedical Engineering, Faculty of Mechanical Engineering, Bialystok University of Technology, Wiejska 45C, 15-351 Bialystok, Poland; anna.powojska@sd.pb.edu.pl; 2 Department of Automatic Control and Robotics, Faculty of Electrical Engineering, Bialystok University of Technology, Wiejska 45D, 15-351 Bialystok, Poland; a.mystkowski@pb.edu.pl; 3 Department of Thermal and Energy Engineering, School of Mechanical Engineering, Vellore Institute of Technology (VIT), Vellore 632 014, India; edison.g@vit.ac.in

**Keywords:** surface modification, spin-coating, magnetic composites, biocompatible layer

## Abstract

This paper verified the possibility of applying chitosan and/or ferulic acid or polycaprolactone (PCL)-based coatings to polydimethylsiloxane/neodymium–iron–boron (PDMS/NdFeB) composites using the spin-coating method. The surface modification of magnetic composites by biofunctional layers allows for the preparation of materials for biomedical applications. Biofunctional layered magnetic composites were obtained in three steps. The spin-coating method with various parameters (time and spin speed) was used to apply different substances to the surface of the composites. Scanning electron microscopy (SEM) and confocal laser scanning microscopy (CLSM) were used to analyze the thickness and surface topography. The contact angle of the obtained surfaces was tested. Increasing spin speed and increasing process time for the same speed resulted in decreasing the composite’s thickness. The linear and surface roughness for the prepared coatings were approximately 0.2 μm and 0.01 μm, respectively, which are desirable values in the context of biocompatibility. The contact angle test results showed that both the addition of chitosan and PCL to PDMS have reduced the contact angle *θ* from 105° for non-coated composite to *θ*~59–88° depending on the coating. The performed modifications gave promising results mainly due to making the surface hydrophilic, which is a desirable feature of projected biomaterials.

## 1. Introduction

Silicone-based composites reinforced with NdFeB magnetic particles are a developing group of materials. Elastic magnetic composites are widely used in automatics and electronics due to their unique properties. There is a growing interest in using these multifunctional materials as smart materials in biomedical engineering applications, i.e., for endoscopic capsule robots, sample manipulation on lab-on-chip systems, drug delivery systems, and microsurgery [1]. However, NdFeB-based elastic composites without surface modification may cause adverse reactions and even be toxic. Thus, this requires the biofunctionalization of these materials to protect them against corrosion, which occurs during the release of magnetic powder particles into the environment. The surface is also expected to prevent biofilm growth and exhibit antibacterial properties. Our previous studies showed that the properties of tested materials and 0.9% NaCl solutions after incubation were changed as a result of a materials’ corrosion [2].

Surface modification is one of the methods of changing the biological and physiochemical properties of the material. The functionalization of the surface should provide anti-corrosion protection and ensure the stability of the material surface properties after incubation [3,4,5]. There are many known ways to functionalize and change the properties of the PDMS surface, including modification with the addition of silane, e.g., (3-Aminopropyl)triethoxysilane (APTES), plasma treatment, and ultraviolet (UV) radiation [1]. Unfortunately, due to the nature of the PDMS/NdFeB composite, surface modification using those methods is not sufficient. However, changes in the properties of these composites are not the only problem. The physical or chemical protection against the release of NdFeB particles into the biological systems is also important. Therefore, it seems necessary to prepare coatings that will have a positive effect on the surface properties and will constitute a mechanical barrier against particle release in a working environment.

The production of soft materials for medical applications is challenging due to the combination of physicochemical, mechanical, and biological properties. One of the challenges is creating a biocompatible and bioactive surface [6], so many factors are considered during the design of biomaterials. In the case of insufficient properties, the material is subjected to chemical and physical modifications. One type of modification is a surface functionalization of the material, which is achieved using another substance that will demonstrate good adhesion to the substrate and will also ensure that certain properties are changed in the desired direction. Thin, flexible materials are produced by various techniques. Flat-layer composites with a thickness of 10–200 μm can be produced by pressing, sintering, chemical/physical vapor deposition, or by using 3D printing technology [1,7,8]. The production method depends mainly on the type of applied coating.

In the case of elastic polymers, the number of manufacturing options is limited, and one of the best techniques is the spin-coating method. The spin coating process enables precise control over the film thickness and offers uniformly dispersed coatings [9]. The covering substrate, which is dispersed on the material’s surface, is in a liquid or, eventually, sol-gel form. The deposited material is usually dissolved in a solvent that is removed during the spin-coating process [10]. One of the factors determining the final layer of thickness is the centrifugation speed [10,11]; thus, the spin coating process is controlled by the spin speed, usually measured in rpm. The radial force exerted on the fluid causes the fluid to shear toward the edges of the substrate, and the liquid is driven outwards by the centrifugal force. Then, surface tension combined with the fluid shear force causes a thin film formation on the flat surface of the material [12]. It may also happen that the curing and cross-linking process, especially in the case of self-curing polymers, occurs sometime after the spin coating process is completed. Solvent evaporation is also an important factor because the viscosity of the solution increases, and the movement is hindered. The samples require additional time for chemical bonding that is combined with solvent evaporation. Additional processing steps may also be used in the process, e.g., curing by UV radiation [13]. There are some defects associated with the spin coating process, such as comets, striations, or chuck marks, described elsewhere [13,14]. It should also be taken into account that spin coating is not suitable for all materials, due to their viscosity or other limitations [6]. In the case of coatings applied to materials for medical applications, not only the method of coating is important, but also the selection of a substance that must be biocompatible. Based on the literature analysis and preliminary analysis performed by the authors of this work, three chemical substances were selected for coating PDMS/NdFeB composites: chitosan (CHIT) [10,11]; ferulic acid (FA) [12,13]; and polycaprolactone (PCL) [9,14]. Chitosan, derived from chitin, exhibits good biocompatibility and biodegradability and is widely used in various biomedical applications, such as wound healing, drug delivery, and tissue engineering [15]. Chitosan nanoparticles are used for drug delivery in medicine, as well as in the context of biodegradable packaging in the food industry [16]. Studies indicate that chitosan, due to its natural origin, promotes cell adhesion and proliferation and is characterized by anti-microbial properties [17], preventing bacterial adhesion and biofilm formation on surfaces [14]. However, the biocompatibility of chitosan can be influenced by factors such as the degree of deacetylation, molecular weight, and specific application conditions [10]. Chitosan is used in pharmacy as a targeted therapy for cancer tumors and for gene delivery, which are very effective treatment methods [18]. Chitosan is also widely used in active food packaging due to its ability to maintain the freshness of products and show antioxidant activity [19,20]. Chitosan is the basis of films created for food packaging, maintaining mechanical stability and beneficial antibacterial properties [21] and, as a biopolymer, is also used for environmental purposes and employed in clarification, water purification, wastewater treatment, remediation, sludge dewatering, and membrane filtration [22]. Chitosan, together with ferulic acid, is used to make biodegradable films, which constitute a barrier to oxygen when packaging food in an ecological way [23]. PCL is a biodegradable polyester and, due to its biocompatibility, slow degradation rate, supporting cell adhesion, proliferation, differentiation, and minimal inflammatory response, is used in the field of tissue engineering, drug delivery [14,24], and long-term implants [25]. Kim et al. [26] describe the use of antibacterial polycaprolactone on scaffolds in regenerative medicine. Espinoza et al. [27] describe the use of PCL in the treatment of various types of cancer. This polymer is used in modern treatments, including nanomedicine. PCL, together with other polymers, including PEG and PLA, is a drug delivery agent [28,29]. This polymer is also used as a component of hydrogels for 3D printing and creating artificial cartilage and thermosensitive materials [30,31,32]. The biocompatibility of PCL can be influenced by many factors, e.g., molecular weight, processing methods, and specific application conditions [33]. PCL also works well in sustainable agriculture, where it is used in the form of mulching foil or composites [34]. The advantage of this solution is the nanoporosity of the biodegradable polymer structure, which can be obtained during production [35]. Ferulic acid is a natural compound found in various plants; it is known for its antioxidant properties and is commonly used in skincare products, often in combination with vitamins C and E [12]. In terms of biocompatibility, ferulic acid is generally considered safe and well-tolerated by the human body [36]. Some studies have explored its potential therapeutic effects, including anti-inflammatory and anti-cancer properties, as well as its contribution to cardiovascular health by reducing oxidative stress and inflammation, which are factors associated with heart diseases [37]. Ferulic acid encapsulated in the form of nanoparticles can be used in drug delivery, crossing the blood–brain barrier [38]. Turkez et al. [39] report that ferulic acid is a promising substance for the treatment of Alzheimer’s disease. As an antioxidant, ferulic acid may help neutralize free radicals, which are implicated in various diseases, including neurodegenerative disorders [40]. Cavalcanti et al. [41] indicate the wide use of ferulic acid as an ingredient in cosmetics and dermocosmetics, being responsible for proper hydration and reconstruction of the skin. It has been shown that the use of cosmetics based on ferulic acid slows down the visible aging processes [12].

To the best of our knowledge, there are no publications on the modification of elastic magnetic composites with biocompatible flexible layers. This work aimed to verify the possibility of applying coatings on elastic PDMS/NdFeB magnetic composites using the spin-coating method and examination of the influence of the coating type on selected material properties. The original chemical composition of the coatings was used, and the process parameters were selected experimentally.

## 2. Materials and Methods

Elastomeric composites coated with three different types of coatings were tested in this work. Firstly, the PDMS/NdFeB composites were prepared and then coated by PDMS combined with chitosan or chitosan and ferulic acid or polycaprolactone of different molecular weights, using the spin-coating method. For prepared materials, microscopic observations, surface roughness analysis, tensile tests, and water contact angle measurements were performed.

### 2.1. Materials

The following chemicals were used to prepare composites and coatings:

- Sylgard 184 elastomer (Dow Corning, Midland, MI, USA);
- NdFeB microparticles (MQFP-14-12-20000-088, Magnequench, Singapore);
- Low molecular weight (LMW) chitosan, Mw~50,000 (Pol-Aura, Olsztyn, Poland);
- Ferulic acid (FA), Mw = 194.18 (Pol-Aura, Olsztyn, Poland);
- Polycaprolactone (PCL) with two different molecular weights: Mw~14,000; and Mw~80,000 (Sigma-Aldrich, Steinheim, Germany).

### 2.2. Composite Preparation

**Figure 1:**
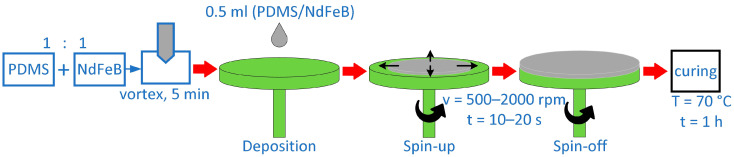
Preparation of PDMS/NdFeB composites using spin-coating method.

NdFeB microparticles were used as a magnetic filler, and Sylgard 184 was used as a polymer matrix to create PDMS/NdFeB composites. The MQFP-14-12 micropowder’s composition is listed elsewhere [33]. The materials were prepared using 50% silicone resin by weight and 50% magnetic powder by weight in order to obtain the required material qualities. As per the manufacturer’s instructions [34], the silicone elastomer was first combined with the curing agent in a 10:1 ratio. NdFeB particles were then combined and homogenized with the liquid elastomer. As per this study [33], the produced mixture was agitated for 5 min to achieve a homogeneous dispersion of magnetic powder in the silicone matrix. According to the diagram in Figure 1, the liquid composite was utilized to prepare thin samples using the spin-coating technique after the homogenization process. Every 250 rpm, the spin speed that was employed to spin-coat the composites changed between 500 and 2000 rpm. The single process took between 10 and 20 s, increasing every 5 s. An even layer of the coating liquid, 0.5 mL in volume, was applied to a 60 mm-diameter Petri dish surface. The procedure of spin-coating was carried out under room temperature (21 ± 1 °C) and humidity (70 ± 5%). The final phase required the cross-linking of the silicone elastomer in the composite, which took place in a laboratory drying–heating chamber (Binder GmbH, Tuttlingen, Germany) [42] at a temperature of 70 °C for one hour.

### 2.3. Coatings Preparation

The compositions of the coatings were determined experimentally, based on the literature data [13,43,44,45] and our own experience. The main factor determining the selection of the coating was the desired biocompatibility and expected antibacterial properties of the obtained surface-modified magnetic composites [10,46,47,48]. Different coatings were applied to PDMS/NdFeB composites.

**Figure 2:**
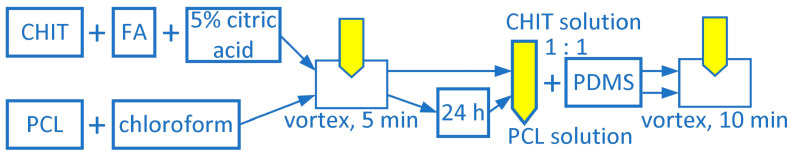
Scheme of preparation of CHIT/FA and PCL coatings.

In the first group, there were five coatings, such as PDMS with the addition of different concentrations of chitosan and PDMS with the addition of chitosan and ferulic acid. The scheme for preparing solutions for coatings is presented in Figure 2.

A solution containing five percent citric acid (Sigma-Aldrich, Steinheim, Germany) was used to dissolve the proper concentrations of chitosan and ferulic acid. Using a vortex stirrer, the solutions were stirred for five minutes in order to homogenize them [42,43]. Four PDMS coatings with varying molecular weights of PCL were included in the second group. The proper quantity of PCL was dissolved in chloroform to create these solutions. For five minutes, each solution was stirred with a vortex stirrer. The solutions were then allowed to dissolve completely in the solvent overnight at room temperature in closed glass flasks due to the rather lengthy dissolution procedure [44,45]. Following was the process used to prepare the final homogeneous coating solutions: one part of PDMS was combined with one part of either (a) polycaprolactone solution (PDMS-PCL) or (b) chitosan or chitosan plus ferulic acid solution (PDMS-CHIT). A pure PDMS solution was used as the reference. Table 1 displays the chemical compositions of the coatings that were applied.

### 2.4. Preparation of Layered Composites Using Spin-Coating Method

**Figure 3:**
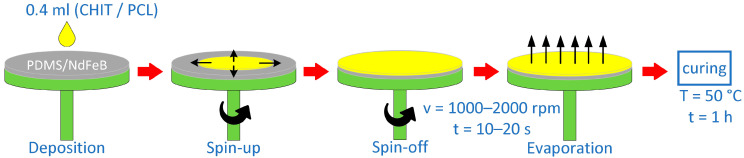
Preparation of CHIT/FA or PCL coatings on the surface of PDMS/NdFeB composite using spin-coating process.

After preparing the liquid coatings, the PDMS/NdFeB composites were subjected to a spin-coating process according to the scheme presented in Figure 3. The final materials were prepared by applying a coating layer to a composite surface and spreading uniformly over the surface using various process parameters. This process was conducted using a POLOS SPIN150i spin-coater (SPS International, Oude-Tonge, The Netherlands). Process parameters were optimized based on the earlier tests and the literature data. The main factors considered were the chemical composition of the coatings, their viscosity, and the expected coating thickness. The device control allows us to set the rotation speed with an acceleration of up to 1000 rpm, an accuracy of 10 rpm, and the time with an accuracy of 1 s. In this work, the speed used for the spin-coating varied between 1000 and 2000 rpm, changing every 500 rpm. The coating time was in the range of 10–20 s, increasing every 5 s. A constant volume of 0.4 mL of the coating liquid was distributed evenly on a flat PDMS/NdFeB surface with a diameter of 60 mm. The spin-coating process was realized under room conditions of temperature (21 ± 1 °C), humidity (70 ± 5%), and pressure (100 ± 10 kPa). The process carried out under the conditions described above allowed for obtaining a layer with a thickness of 60–100 µm. The composite matrix resin, i.e., PDMS, is incorporated into a solution containing either PCL in chloroform or chitosan in citric acid in a 1:1 volume ratio. The coating solution is homogenized on a vortex stirrer for 10 min. This process was repeated on both sides of the thin composite. After the spin-coating process, the samples were dried in an incubator for 1 h at 50 °C. The processes of covering the PDMS/NdFeB composite by biocompatible layers of PDMS-CHIT/FA or PDMS-PCL were described in detail in the authors’ patent applications P.445106 [49] and P.445105 [50].

Materials’ characterization included the microscopic observations (CLSM, SEM) of the cross-section and surface of modified composites, which were performed to confirm the proper application of coating using the spin-coating method. The coatings’ thicknesses (*h*) and the water contact angles (*θ*) of the obtained coatings were also measured.

### 2.5. Thickness Measurements

The Phenom XL desktop scanning electron microscope (SEM) (Thermo Fisher Scientific, Waltham, MA, USA), according to the ISO 9220 standard [51], was used to measure thin film thickness. The samples were cross-sectioned to reveal the arrangement of layers and their thicknesses. The samples were placed in the microscope chamber on a stand and a holder that allowed for the sample to be held in a vertical position. Each time, the cross-section of the sample was assessed, including the measurements of the core composite film and the coating thickness. This process was repeated three times for each material. Based on the obtained results, the mean value and standard deviation of the measurements were calculated.

### 2.6. Surface Roughness Measurements

The evaluation of surface roughness was performed using Olympus LEXT OLS 4000 Confocal Laser Scanning Microscope (CLSM) (Olympus, Tokyo, Japan), according to the ISO 25178-607 standard [52]. The surfaces of samples were firstly cleaned with ethanol and ultrapure water to avoid imaging any contamination. The non-destructive method with real-time imaging gives a three-dimensional representation of the surface using both laser and white light. The Ra and Sa parameters were calculated using microscope software (LEXT OLS4000 software, version 2.2). For each sample, five measurements were taken. Based on the obtained results, the mean value and standard deviation of the measurements were calculated [53].

### 2.7. Water Contact Angle Measurements

The Contact Angle Goniometer (Ossila, Sheffield, UK) was used to measure the contact angle (*θ*) of water on the examined samples. The measurements were performed according to the ISO 19403-2 standard [54] and the Zisman method [55]. Each measurement consists of pouring a 5 µL droplet of deionized water (Milli-Q) on a substrate surface and measuring the contact angle immediately after placing the droplet [56]. The values were calculated using dedicated software (Ossila Contact Angle Goniometer software version 4.0). The tests were repeated five times for each sample. The mean value and standard deviation were then calculated. The water contact angle test determined the surface wetting characteristics [6].

Additionally, contact angle measurements were performed for ethanol and acetone. Based on the surface contact angle results, the surface tension of each material was calculated. The Zisman model was used for this purpose [57,58]. The assumed surface tension values were water (72.75 mN/m), ethanol (22.31 mN/m), and acetone (23.24 mN/m) at a temperature of 20 °C [59,60]. Based on the fitted trend line and linear equation, the surface tension value was calculated, for which cos *θ* = 1.

### 2.8. Density Measurements

For density tests, pure coating samples were prepared by applying each coating solution on the surface of the Petri dish. After evaporation of the solvent, density tests only for coatings were performed. The density (*d*) of the prepared coatings was measured according to the ISO 1183-1 standard [61], using the balance (Mettler Toledo, Columbus, OH, USA) with the system dedicated to the hydrostatic method. It allows us to measure the density of solid items, first by weighing and then by submerging the sample in a container filled with deionized water (Milli-Q). The density was calculated automatically by the included software [56]. This method is relatively simple and requires minimal equipment added to the balance, making it a convenient technique for density measurements. The tests were repeated five times for each sample. The mean value and standard deviation were then calculated.

### 2.9. Tensile Tests

Tensile strength tests of elastomers were performed on the SAUTER Test Stand machine (Fr. SAUTER AG, Basel, Switzerland). The FH 20 force gauge sensor was used for the tests. The maximum measurement capacity is 20 N, and the resolution is 0.01 N. The tests were carried out on rectangular samples with a thickness of up to 500 μm, a width of 10 mm, and a distance between the grips of 50 mm. The test was set up according to ISO 527-3 standard [62], with a velocity of 100 mm/min. The tests were repeated five times for each sample. The mean value and standard deviation were then calculated.

### 2.10. Statistical Analysis

The statistical analysis was performed using Statistica software (TIBCO Statistica^®^ software version 14.0.1, Palo Alto, CA, USA). Based on the results obtained from at least five repeatable test attempts under the same conditions, the average value and standard deviation were calculated. The results are presented as mean value ± SD.

## 3. Results and Discussion

The experimental part of this work included conducting the spin-coating process, where adjusting the process parameters of preparation of PDMS/NdFeB composites, chitosan/PCL coatings, and evaluating the properties of obtained functionalized layer composites were performed.

### 3.1. Preparation of Layered Composites Using Spin-Coating Method

The spin-coating process was used to create the PDMS/NdFeB composites in the first step. The spin speed and spin time were measured in rpm and s, respectively. Table 2 illustrates how decreasing the composite’s thickness was the outcome of increasing spin speed as well as increasing process time at the same speed. The thickness of the PDMS/NdFeB composites formed with 500, 1000, and 2000 rpm spin speeds and process duration of 10 s was 317.9 µm, 216.3 µm, and 97.4 µm, respectively, according to the results. The thickness fell by 30–35% or 50–55%, respectively, when the spin speed was increased from 500 to 1000 rpm or from 1000 to 2000 rpm at the same process time. Considering the processing time, altering it from 10 to 15 s or from 15 to 20 s results in a similar outcome of a 20–25% reduction in thickness at one speed. The comparatively low standard deviation values show that the magnetic composite thickness (h) measurements were repeatable. The actual process of spin-coating is repeatable, and human intervention during further, repeated attempts has minimal effect. The spin-coating process was used to create the PDMS/NdFeB composites in the first step. The spin speed and spin time were measured in rpm and s, respectively. Table 2 illustrates how decreasing the composite’s thickness was the outcome of increasing spin speed as well as increasing process time at the same speed. The thickness of the PDMS/NdFeB composites formed with 500, 1000, and 2000 rpm spin speeds and process duration of 10 s was 317.9 µm, 216.3 µm, and 97.4 µm, respectively, according to the results. A 30–35% or 50–55% reduction in thickness was achieved by raising the spin speed from 500 to 1000 rpm or from 1000 to 2000 rpm at the same process time. Considering the processing time, altering it from 10 to 15 s or from 15 to 20 s results in a similar outcome of a 20–25% reduction in thickness at one speed. The comparatively low standard deviation values show that the magnetic composite thickness (h) measurements were repeatable. The actual process of spin-coating is repeatable, and human intervention during further, repeated attempts has minimal effect.

**Figure 4:**
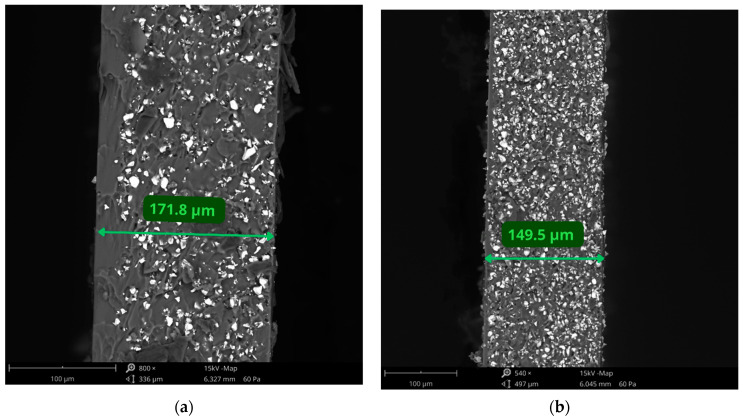
SEM analysis of PDMS/NdFeB composites prepared using the spin-coating method. (**a**) v = 1250 rpm, t = 10 s and (**b**) v = 1500 rpm, t = 10 s; scale bar = 100 μm.

Figure 4 shows two of the obtained PDMS/NdFeB composites prepared by the spin-coating method, using the following parameters: (a) v = 1250 rpm, t = 10 s; and (b) v = 1500 rpm, t = 10 s. The SEM analysis showed that the thickness of the composites obtained with these parameters were 171.8 µm and 149.5 µm, respectively. The microscopic analysis also shows that magnetic particles are well-dispersed in a silicone matrix. It is also possible to determine the size of magnetic particles, which, according to the manufacturer, is d_50_ = 25 µm.

A composite with a thickness of approximately 150 μm was selected. The composite with this thickness was created using the following process parameters: v = 1500 rpm; and t = 10 s, in preparation for additional testing and the coating procedure. Using the parameters listed in Table 3, coatings were applied to the composites that were created in this manner in the second phase. Comparable coating thicknesses from various solutions were made possible by adjusting the coating deposition time for solutions with varying viscosities and setting the rotational speed between 1000 and 2000 rpm. The coatings that were deposited on the surface had a thickness of roughly 80–100 µm. Time and spin speed determined the multilayer flexible magnetic composite’s thickness. It has been confirmed that coatings made of pure chitosan or PCL are inappropriate for use on elastic materials. The poor adhesion to the silicone surface and crumbling of the coatings due to high stiffness (higher than for silicone composite) were observed. Furthermore, the coatings acted like glass following application and evaporation, flaking and cracking off the surface they were applied to. As a result, the authors of this work tried to increase the coating’s flexibility while maintaining its elasticity and PDMS adherence. To find the ideal parameter setting that would allow for the production of layers with comparable thickness, the spin-coating process was run under a number of different parameter values. In addition to measuring the thickness h, the spin speed v and time t were changed. The factors that allowed for the production of coatings with a thickness of less than 100 μm are displayed in Table 3.

As can be seen in Table 3, the process parameters for spin-coating tested solutions were different, which was related mainly to the different viscosity of the applied solutions. From the prepared solutions with specific chemical compositions (as shown in Table 1), chitosan solutions showed higher viscosity compared to solutions containing PCL, which resulted in poorer distribution of the chitosan solutions on the composite surface. Chitosan coatings required a higher speed (e.g., 2000 rpm and 10 s) or longer time (e.g., 1500 rpm and 15 s) to obtain thicknesses like those obtained with PCL (where only 1000 rpm and 15 s or 1500 rpm and 10 s are sufficient). Additionally, the higher the chitosan or PCL concentration in the PDMS mixture, the higher the required spin-coating speed and process time. For example, for PDMS-CHIT1, 1500 rpm and 10 s were required to obtain 101.6 µm, while for PDMS-CHIT3, 2000 rpm and 15 s were required to obtain 98.0 µm. The addition of ferulic acid also resulted in poorer spreading of the coating compared to an equivalent solution with chitosan without the presence of ferulic acid. In the case of PCL, the higher molecular weight of the polymer required a higher speed or longer time in the spin-coating process. The PDMS-PCL 14-10 sample needed 1000 rpm and 15 s, while PDMS-PCL 80-25 required 1500 rpm and 10 s to obtain a thickness of around 100 µm. The polycaprolactone spreading had to be performed in the shortest possible time and, therefore, at higher speeds due to the rapid evaporation of chloroform [63]. The tested process parameters made it possible to obtain chitosan or PCL coatings on the surface of the PDMS/NdFeB composite with repeatable thickness. As a result, layered composites made of chitosan or PCL coatings with good adhesion to the surface of the hydrophobic PDMS/NdFeB composite were obtained. It was observed that for tested chitosan/PCL solutions, spreading the same amount of material in liquid form over a surface at a higher speed or for a longer period resulted in a lower thickness. It can be concluded that the determination of the theoretical coating thickness is possible if the properties of the liquid substance being applied are known [64,65].

Table 4 presents the thickness results of two coatings for which the spin-coating process was carried out most effectively, allowing for uniform coatings to be achieved on the composite surface. The first one was the PDMS-CHIT2 coating, and the second one was the PDMS-PCL 14-10 coating. The selected coatings were analyzed more broadly because they were easy to spread, and they resulted in an evenly distributed layer. For chitosan-based solutions, their high viscosity caused difficulties during the process. At a concentration of 3% chitosan or for the solutions with the addition of 1% FA, it was difficult to spread the coating. In the case of PCL coatings, the rapid evaporation of the chloroform solvent from the coating solution was challenging. At a higher PCL concentration in the solution and for solutions with higher molecular weight of PCL, carrying out the spin-coating process was problematic.

**Figure 5:**
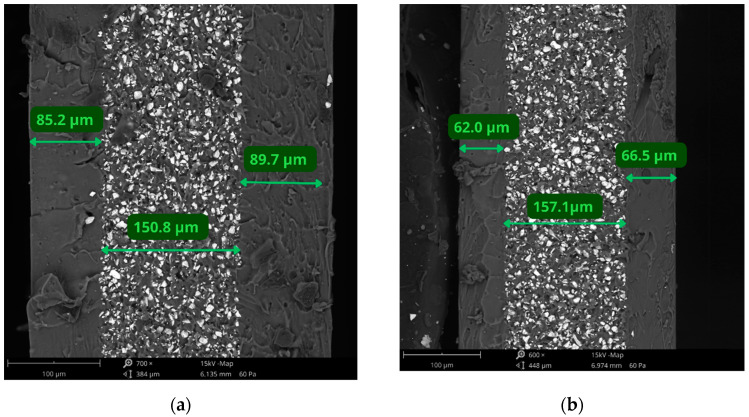
The cross-section of (**a**) PDMS-CHIT2. (**b**) PDMS-PCL 14-10 coated samples prepared using the spin-coating method; scale bar = 100 μm.

Results in Table 4 show that for the same parameters, completely different coating thickness is obtained for solutions with different chemical compositions. The PDMS-CHIT2 coating consists of PDMS mixed with 2% chitosan solution in citric acid. The PDMS-PCL 14-10 consists of PDMS mixed with 10% PCL solution in chloroform. The PCL molecular weight is around 14,000. For the same solution concentrations and spin-coating parameters, the chitosan-based coating is thicker than the PCL-based coating. For example, for spin-coating with the spin speed of 1000 rpm for 15 s, the thickness of PDMS-CHIT2 coating is 202.7 µm, and for the same parameters for PDMS-PCL 14-10, it is 100.9 µm. The changes are proportional, just like in the case of PDMS/NdFeB composites. Examples of coating cross-sections are shown in Figure 5. The PDMS-CHIT2 coatings prepared using 2000 rpm for 20 s (Figure 5a) and PDMS-PCL14-10 coatings prepared by using 1250 rpm for 20 s (Figure 5b) were measured and analyzed. In the middle of the sample, the elastic composite can be seen.

Due to the different chemical composition of each solution, primarily the different viscosity of the substances, it is necessary to verify the thickness for each substance separately. When preparing the solutions, the difference in the viscosity of the solutions can be noticed. The spin-coating process allowed us to obtain a thickness of about 30–350 μm, depending on the spin speed and the type of deposited solution.

It was expected that a higher concentration of the same substance in solution or a higher molecular weight of the dissolved substance would result in a higher viscosity of the dispersed solution. Therefore, it was predicted that the substance would be more difficult to spread at higher concentrations, as well as obtain a lower thickness for solutions with lower concentrations of the active substance. The process parameters should be adjusted each time, depending on the solution used, due to the different viscosities and densities of the substances applied.

### 3.2. Linear and Surface Roughness

**Figure 6:**
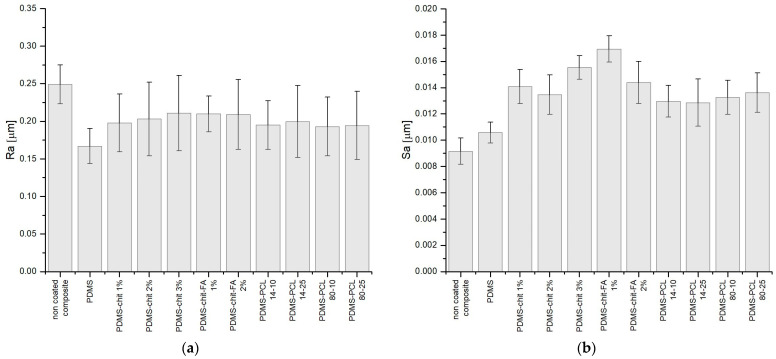
The surface topography results: (**a**) linear roughness; (**b**) surface roughness for the examined coatings.

Features like ridges, grooves, or scratches that are lined in a certain direction on the measured surface are indicative of linear roughness. A surface’s linear roughness has an impact on adhesion, wear, and friction. Linear roughness in biomaterials affects the adhesion, alignment, and migration of cells [62]. Protein adsorption, cellular reactions, and the general behavior of biological tissues can all be impacted by surface roughness [63]. It is possible that smoother surfaces encourage improved cell adhesion, which is crucial for tissue integration. The average roughness Ra parameter is typically used to quantify linear roughness. The values obtained for linear roughness *Ra* are presented in Figure 6a. The highest linear roughness was observed for non-coated composite (*Ra*~0.249 µm). The obtained values for the coatings with additives (*Ra*~0.217 µm for chitosan-based coatings and *Ra*~0.198 µm for PCL-based coatings) are relatively lower than for non-coated composite. The PDMS coating is the least rough of all applied coatings, with a roughness of *Ra*~0.167 µm. This means that the used coatings allowed for reducing the linear roughness of the surface. No significant differences in roughness results were observed, considering the different additives contained in the coatings.

It should be noted that the obtained roughness values are appropriate from the point of view of biocompatibility, as indicated by the literature reports. Fadzil et al. [66] pointed out that the roughness of machine-made implants, with a value of 2.15 μm, is even a hundred times lower than that of traditionally made, sandblasted materials, which is a favorable result in the context of biocompatibility. According to Yeniyol et al. [67], a *Ra* roughness value of 4 μm or 30 μm does not adversely affect cell adhesion and antibacterial properties, but it is important to keep *Ra* at a certain low level. Authors of this work also indicate that even *Ra* = 30 μm may bring a beneficial effect in combination with other parameters.

The obtained values of surface roughness *Sa* are presented in Figure 6b. The obtained roughness differs for non-coated samples (*Sa*~0.00924 µm) and for those containing coatings. The obtained values for the coatings with additives (*Sa*~0.0146 µm for chitosan-based coatings and *Sa*~0.0131 µm for PCL-based coatings) are relatively higher than for non-coated composite. Samples of clean, non-coated composite have the lowest roughness. The PDMS coating is the least rough of all applied coatings (*Sa*~0.0179 µm). Higher roughness values were observed for chitosan-based coatings in comparison to PCL-based coatings. However, it should be noted that in all cases, the roughness is not higher than 0.02 μm, which is a satisfactory result, allowing us to conclude that a deviation of the value by a few thousand μm will not have a significant impact on the surface parameters in the context of the material’s use [68,69]. Much higher roughness values of biomaterial surfaces are reported in the literature. For metallic materials, Quirynen et al. [68] have reported, using in vivo studies, that surface roughness below 0.2 μm does not affect bacterial adhesion. In the case of the composites and coatings tested in this work, the surface’s roughness value was one order lower. For resins, the results presented by Lee et al. [69] were not so promising, as bacterial growth was observed on material with a roughness lower than 0.2 μm. The response of microorganisms to the material must, therefore, be examined to assess its usefulness in medical applications. Previous research [43,70] shows that not only roughness but also the type of material is important.

**Figure 7:**
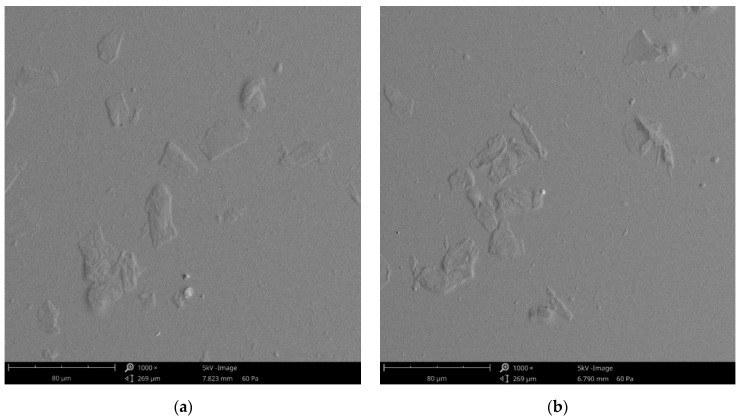
SEM analysis of the composite samples covered with (**a**) PDMS-CHIT2 coating and (**b**) PDMS-PCL14-10 coating; scale bar = 80 μm.

The coatings topography was measured using a confocal laser microscope, and microscopic observations were carried out using SEM microscopy. In Figure 7, the field emission scanning electron microscope images of the two surfaces can be seen, covered with a PDMS-CHIT2 coating (Figure 7a) and a PDMS-PCL14-10 coating (Figure 7b). Obtained images show that there are no significant differences in the coatings. The particles visible in the figures are parts of the composites because the coatings are somewhat transparent.

### 3.3. Contact Angle

**Figure 8:**
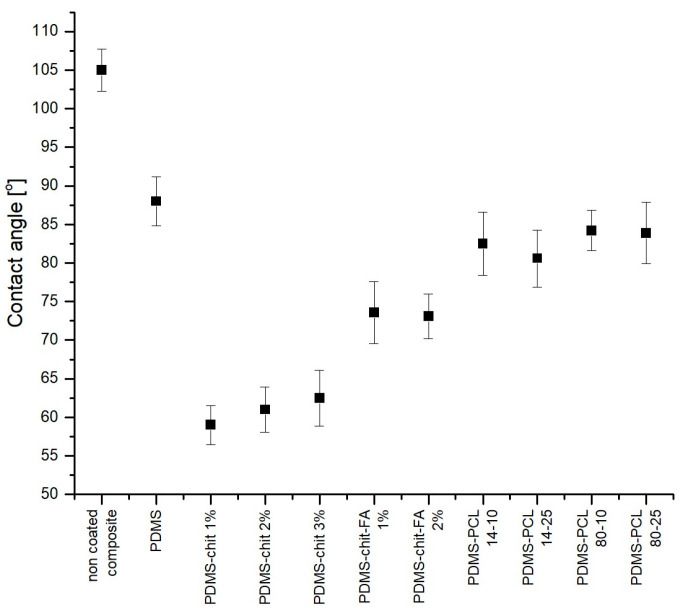
The water contact angle values of the examined sample’s surface.

The measurement of the biomaterials’ contact angle makes it possible to determine the wettability of the material’s surface. It is a crucial parameter in understanding the wettability of biomaterials, influencing processes like cell adhesion and protein absorption. A higher contact angle, above 90°, indicates hydrophobicity, while a lower angle (below 90°) indicates hydrophilicity. Tailoring the water contact angle value, which is strictly connected with the wettability of the surface, is essential in designing biomaterials for specific applications where interactions with biological fluids play a critical role. The contact angle measurement results are shown in Figure 8. The water contact angle for the pure PDMS coating is *θ*~88°, which means that the surface tends to be hydrophilic. When PDMS is mixed with the metal powder to obtain the composite, the contact angle for that surface increases to about *θ*~105°, which can be classified as a hydrophobic surface. The water contact angle of PDMS-PCL coating (e.g., for PDMS-PCL 14-10 *θ* = 82.6° ± 4.1°) is similar to the contact angle of pure PDMS (*θ* = 87.3° ± 2.8°). This may indicate a similar chemical and physical nature of PDMS itself and PDMS with the addition of PCL. The bonds formed and reactions occurring in the mixture do not affect the final surface energy. For PDMS-CHIT coatings, the content of additives reduces the contact angle (*θ* = 59.1° ± 2.9° for PDMS-CHIT1). This may suggest that the bonds formed between PDMS, chitosan, and ferulic acid influence the change in the energy state of the coating and, consequently, affect the wettability of the surface. The higher the chitosan concentration, the greater the contact angle reduction. The contact angle of the biofunctional layers was lower (*θ_c_* = 71.1 ± 3.8°) compared to the PDMS/NdFeB powder composite surface (*θ_c_* = 104.8 ± 2.7°).

Functionalization of a surface by using chitosan solutions causes a significant reduction in the surface wetting angle. The value is in the range of *θ* = 59~63°, which means that it is 25–30° lower than the value of this angle for pure PDMS. No significant change in the contact angle was observed depending on the chitosan concentration in the coating. The presence of both chitosan and FA results in the contact angle of *θ* = 73°, so the addition of FA leads to an increase in the contact angle. Compared to the reference sample (pure PDMS), the value is 15° lower for coatings with chitosan and FA. For surfaces made of material with the addition of PCL, the contact angle value is slightly lower than for pure PDMS. The value of the contact angle is in the range *θ* = 81~84°. It has been noted that as the PCL concentration increases, the contact angle decreases slightly. The lower molecular mass of PCL added to the coating solution also caused the reduction in contact angle. The contact angle is, on average, 3° lower for the coatings with lower PCL molecular mass. Changing the contact angle to a lower one results in greater hydrophilicity of the material. This affects cell proliferation and may also prevent proteins from attaching. Both of these phenomena are considered beneficial from the point of view of applications in medicine [71,72].

The surface tension γ for coatings was calculated and compared to the surface tension of the PDMS/NdFeB composite (γ = 21.83 mN/m). Data obtained from the analysis are presented in Table 5.

The surface tension for pure PDMS coating is slightly lower (γ = 20.12 mN/m) than the surface energy of the composite. These data are similar to the literature data, which report a surface tension of 19–21 mN/m for PDMS [73]. Additives to PDMS resulted in an increase in surface energy. PDMS-CHIT1 has a surface tension of 28.28 mN/m, so the addition of chitosan increases this parameter. The addition of ferulic acid also results in a higher surface tension, as the measured value for PDMS-CHIT-FA (γ = 34.12 mN/m and γ = 33.61 mN/m, Table 5) in comparison to PDMS-CHIT1 composite (γ = 28.28 mN/m, Table 5), which gives a difference of 6 mN/m. In the case of coating with the addition of PCL, it was observed that the higher the PCL concentration value, the higher the composite’s surface tension. For coating with 10% PCL and 25% PCL (for which molecular mass was Mw = 14,000), the surface tension values were γ = 28.50 mN/m and γ = 32.69 mN/m, respectively.

Surface tension influences wettability by impacting the equilibrium between adhesive and cohesive forces. Higher surface tension typically leads to the formation of larger contact angles and reduced wettability, as cohesive forces within the liquid take precedence [74]. The phenomenon of lower surface wetting is beneficial in the context of bacterial adhesion. Higher wetting angle and lower wettability support the least amount of transferred bacteria [75,76].

### 3.4. Density of the Coatings

**Figure 9:**
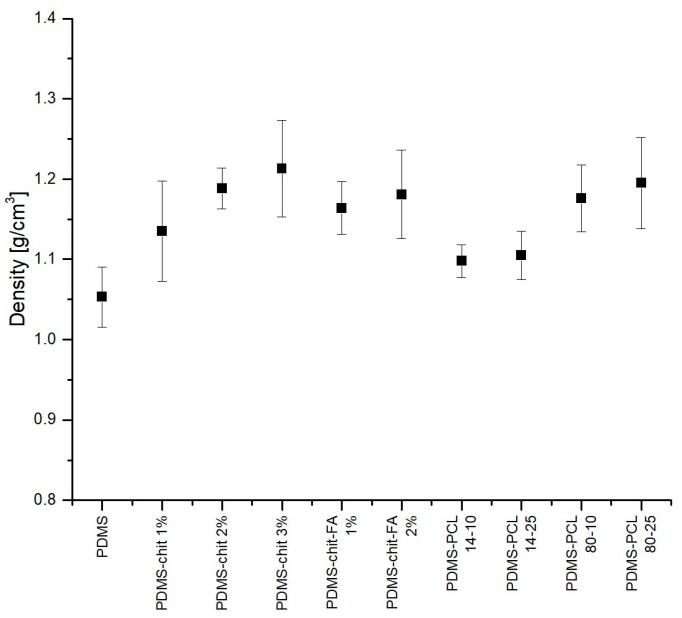
The density of the prepared coatings.

Testing the density of the produced coatings allowed us to determine the cohesion of the material and the impact of a specific coating used on the final weight of the sample. Determining the density is only possible after mixing all the components of the solution, then spreading it on the surface and, in the end, cross-linking the PDMS included in the solution. The measured density is only the density of the coating material after solvent evaporation and cross-linking. The density measurement results are shown in Figure 9.

Pure PDMS has the lowest density, with a density of 1.053 ± 0.037 g/cm^3^. PDMS with functional additives has a higher density, ranging from d = 1.1 to 1.2 g/cm^3^. The higher the concentration of the active substance, the higher the density. For PDMS-CHIT1, the density is d = 1.135 g/cm^3^, while the density for PDMS-CHIT3 is d = 1213 g/cm^3^. For PDMS-PCL 80 with 10% PCL solution, d = 1176 g/cm^3^, and for the coating with 25% PCL solution, d = 1195 g/cm^3^. The density is increased by adding substances with a density higher than pure PDMS. However, after evaporation of the solvent, the density of the entire substance is not much higher than that of PDMS. Many publications [77,78,79,80] mention PDMS as a material for tissue-mimicking phantoms. Goldfain et al. [77] describe the density obtained in these applications, noting that the density will vary depending on the elastomer production method. According to the literature data, the density of PDMS is approximately d = 0.965 g/cm^3^ [81]. In the context of the importance of density in biomedical engineering, it is most often believed that biomaterials with densities similar to those of native tissues may exhibit improved biocompatibility and integration with the host tissue [82]. Also, understanding the density of biomaterials used in drug delivery formulations is essential for optimizing therapeutic efficacy and minimizing adverse effects [10].

### 3.5. Tensile Tests

Tensile tests were performed for the samples, and the results of stress (σ, MPa) of the composites were analyzed. Ultimate tensile strength is the value of the maximum stress that a material can withstand while being stretched before it breaks. For elastomers, tensile strength values depend on the specific elastomer and its intended application. Ariati et al. [83] state that the tensile strength for PDMS is from 3.51 to 5.13 MPa. Johnston et al. [84] indicate that the ultimate tensile strength for PDMS is 3.51–7.65 MPa. It should be emphasized that elastomers demonstrate non-linear stress–strain characteristics, particularly under significant strains. Consequently, their mechanical properties can fluctuate based on the levels of stress or strain applied [85]. Data for the ultimate tensile strength σ, along with the standard deviation, are presented in Table 6.

The initial value of the tensile strength for the composite without coating is very low (*σ* = 0.768 ± 0.176 MPa). The composite with PDMS coating has a maximum stress twice as high as the composite without coating. Even better properties are achieved with coatings with additives. Samples with PDMS-CHIT3 and PDMS-PCL14-25 coatings show approximately six (σ = 4.742 ± 0.197 MPa) and eight (σ = 6.104 ± 0.387 MPa) times higher Young’s modulus. This means that the material obtained after coating will be less susceptible to break under the influence of forces. Based on the results, it can be observed that the tensile strength value does not depend on the process parameters. The addition of chitosan, ferulic acid, and polycaprolactone contributes to higher ultimate tensile strength values. The results obtained for the composite, including the composite with a coating, are lower than those reported in the literature. Johnston et al. [84] measured the ultimate tensile strength of PDMS, and the value obtained was 3.51–7.65 MPa, depending on the curing temperature. Jang et al. [86] investigated the ultimate tensile strength of PDMS and its composites with spherical nickel powder, obtaining a tensile strength of 5.11–8.43 MPa. However, it should be noted that the materials tested in the above work are less than 1 mm thick, which allows them to be classified as foils. However, the behavior of foils is different than that of solid materials.

Overall, it should be noted that obtaining flexible coatings with biofunctional additives opens up new possibilities in the application of materials in medicine. The use of coatings will increase the resistance of the PDMS/NdFeB composite against corrosion. It will also reduce the release of Nd, Fe, or B elements that occurred during incubation in NaCl solution, which was tested and described in our previous work [19]. It will be possible to use materials with coatings on a macro-scale (e.g., for intelligent materials for use on the skin, stimulated by a magnetic field) and on a micro-scale (e.g., for robotic structures for applications in the gastrointestinal tract). Both chitosan, ferulic acid, and PCL are substances with proven antibacterial properties. Further research is planned to actually assess the biological properties of the created material configurations.

## 4. Conclusions

This paper verified the possibility of applying chitosan or PCL-based coatings to PDMS/NdFeB composites. The coatings were obtained on the composites using the spin-coating method. The surface modification of PDMS/NdFeB composite by chitosan and polycaprolactone allows for the preparation of the materials for biomedical engineering applications. The addition of substances with potential antibacterial activity, such as chitosan, ferulic acid, or polycaprolactone, significantly affects the viscosity of coating solutions. This entails the need to individually adjust the parameters each time to obtain coatings of the expected thickness. The thickness of the samples depends strictly on the type of solution. Within the same parameters, the coating thickness values differ even twice. Coatings with the addition of chitosan are thicker than coatings with the addition of PCL, with the same parameters. The thickness decreases as the speed increases and the process time increases. Optimizing surface characteristics, including roughness and contact angle, is a critical consideration in designing biocompatible materials for medical applications. It involves balancing the need for mechanical stability with the desire to encourage positive interactions with biological systems. The roughness value is higher than the PDMS roughness with the addition of chitosan or PCL. The resulting roughness should not, however, have an adverse effect on the biological characteristics of the coatings. The contact angle test results showed that both the addition of chitosan and PCL to PDMS reduces the contact angle value. The higher reduction is noticed for the PDMS-CHIT coatings. The performed modifications give promising results mainly due to making the surface hydrophilic, which is a desirable feature of projected biomaterials. The density of the coatings applied to the composite material has a value that is promoted in many biomedical applications. The PDMS materials, due to their density, are being used as tissue-mimicking materials. Covering the composite with a layer of material has a positive effect on its mechanical properties, especially its tensile strength. The material remains flexible, but it takes several times more force to tear a coated sample than for a non-coated sample. The obtained studies indicate that the surface-modified composite material retains its elastic properties after applying biofunctional layers. However, advanced rheological testing of materials in a controlled magnetic field is needed. The knowledge obtained on a research basis is of practical importance for the use of this type of surface modification for various biomedical applications. The next steps in the research will be focused on two aspects. First is the sterilization and testing of the resistance of the created coatings to specific sterilization methods from the point of view of their use in biomedical engineering. The second is related to conducting biological tests on the material to verify its biocompatible and antibacterial properties, which is planned to be performed in subsequent works.

## Figures and Tables

**Table 1:** Chemical composition of coatings applied on PDMS/NdFeB composites.

Sample	Coating Solution
PDMS	PDMS
PDMS-CHIT1	PDMS + 1 wt.% CHIT
PDMS-CHIT2	PDMS + 2 wt.% CHIT
PDMS-CHIT3	PDMS + 3 wt.% CHIT
PDMS-CHIT-FA1	PDMS + 1 wt.% CHIT + 1 wt.% FA
PDMS-CHIT-FA2	PDMS + 2 wt.% CHIT + 1 wt.% FA
PDMS-PCL14-10	PDMS + 10 wt.% PCL (Mw_PCL_~14,000)
PDMS-PCL14-25	PDMS + 25 wt.% PCL (Mw_PCL_~14,000)
PDMS-PCL80-10	PDMS + 10 wt.% PCL (Mw_PCL_~80,000)
PDMS-PCL80-25	PDMS + 25 wt.% PCL (Mw_PCL_~80,000)

**Table 2:** The thickness of elastic magnetic composites prepared using the spin-coating technique.

v (rpm)	t (s)	h (µm)
500	10	317.9 ± 6.1
	15	263.9 ± 2.8
	20	226.9 ± 6.2
750	10	257.5 ± 2.5
	15	213.7 ± 5.3
	20	185.9 ± 4.4
1000	10	216.3 ± 2.9
	15	181.7 ± 5.0
	20	147.2 ± 6.5
1250	10	175.2 ± 3.8
	15	141.9 ± 3.6
	20	122.0 ± 2.9
1500	10	148.2 ± 4.2
	15	110.7 ± 4.3
	20	94.1 ± 4.1
1750	10	123.3 ± 5.2
	15	104.8 ± 5.6
	20	86.0 ± 2.4
2000	10	97.4 ± 2.6
	15	82.8 ± 3.5
	20	72.9 ± 3.1

**Table 3:** Spin-coating parameters for layered composites preparation modified by chitosan or PCL coating.

Sample	v (rpm)	t (s)	h (µm)
PDMS	1000	10	95.5 ± 3.8
PDMS-CHIT1	1500	10	101.6 ± 5.1
PDMS-CHIT2	2000	10	102.1 ± 3.7
PDMS-CHIT3	2000	15	98.0 ± 4.2
PDMS-CHIT-FA1	1500	20	96.2 ± 5.0
PDMS-CHIT-FA2	2000	20	102.3 ± 3.9
PDMS-PCL14-10	1000	15	100.9 ± 4.5
PDMS-PCL14-25	1000	15	97.8 ± 4.8
PDMS-PCL80-10	1500	10	96.5 ± 3.2
PDMS-PCL80-25	1500	10	101.3 ± 4.4

**Table 4:** The thickness of the chosen coated PDMS/NdFeB composites prepared using different spin-coating parameters.

v (rpm)	t (s)	PDMS-CHIT2 h (µm)	PDMS-PCL 14-10 h (µm)
500	10	343.4 * ± 4.3	159.0 ± 4.0
	15	275.4 * ± 1.9	131.9 ± 2.0
	20	252.9 ± 4.3	113.5 ± 2.5
750	10	268.0 * ± 1.8	128.7 ± 4.0
	15	235.7 * ± 3.7	106.9 ± 3.9
	20	204.4 ± 3.1	93.0 ± 6.5
1000	10	228.3 ± 2.0	108.1 ± 5.3
	15	202.7 ± 3.5	100.9 ± 4.5
	20	174.2 ± 4.5	90.8 ± 4.8
1250	10	191.2 ± 2.7	87.6 ± 6.0
	15	156.9 ± 2.5	71.0 ± 4.5
	20	134.0 ± 2.0	61.0 ± 5.9
1500	10	157.7 ± 2.9	70.1 ± 3.6
	15	128.7 ± 3.0	55.4 ± 3.9
	20	111.1 ± 2.8	47.1 ± 2.8
1750	10	149.1 ± 3.6	61.7 ± 2.8
	15	133.0 ± 3.9	52.4 ± 2.3
	20	110.0 ± 3.3	43.0 * ± 2.8
2000	10	102.1 ± 3.7	48.7 * ± 6.1
	15	97.3 ± 2.4	41.4 * ± 5.8
	20	88.5 ± 4.2	36.4 * ± 3.8

* not uniformly dispersed on the composite surface.

**Table 5:** Surface tension of the obtained coatings.

Sample	γ [mN/m]
PDMS	20.12
PDMS-CHIT1	28.28
PDMS-CHIT2	32.41
PDMS-CHIT3	31.75
PDMS-CHIT-FA1	34.12
PDMS-CHIT-FA2	33.61
PDMS-PCL14-10	28.50
PDMS-PCL14-25	32.69
PDMS-PCL80-10	28.16
PDMS-PCL80-25	30.41

**Table 6:** The ultimate tensile strength of prepared composites.

Sample	v (rpm)	t (s)	σ [MPa]
non coated composite	1500	10	0.768 ± 0.176
PDMS	1000	10	1.609 ± 0.345
PDMS-CHIT1	1500	10	3.204 ± 0.266
PDMS-CHIT2	2000	10	4.083 ± 0.308
PDMS-CHIT3	2000	15	4.742 ± 0.197
PDMS-CHIT-FA1	1500	20	3.367 ± 0.241
PDMS-CHIT-FA2	2000	20	3.608 ± 0.248
PDMS-PCL14-10	1000	15	4.639 ± 0.232
PDMS-PCL14-25	1000	15	6.104 ± 0.387
PDMS-PCL80-10	1500	10	3.508 ± 0.288
PDMS-PCL80-25	1500	10	4.926 ± 0.265

## Data Availability

Data are contained within the article.
